# Automatic Identification of Down Syndrome Using Facial Images with Deep Convolutional Neural Network

**DOI:** 10.3390/diagnostics10070487

**Published:** 2020-07-17

**Authors:** Bosheng Qin, Letian Liang, Jingchao Wu, Qiyao Quan, Zeyu Wang, Dongxiao Li

**Affiliations:** 1 College of Information Science and Electronic Engineering, Zhejiang University, Hangzhou 310058, China; 3170105600@zju.edu.cn; 2 College of Electrical Engineering, Zhejiang University, Hangzhou 310058, China; Letoo147@163.com; 3 College of Computer Science and Technology, Zhejiang University, Hangzhou 310058, China; avada314@foxmail.com (J.W.); 3170104331@zju.edu.cn (Q.Q.); 4 School of Medicine, Zhejiang University School of Medicine, Hangzhou 310058, China; wangzy98@foxmail.com

**Keywords:** deep convolutional neural network, facial recognition, down syndrome, deep learning, facial image

## Abstract

Down syndrome is one of the most common genetic disorders. The distinctive facial features of Down syndrome provide an opportunity for automatic identification. Recent studies showed that facial recognition technologies have the capability to identify genetic disorders. However, there is a paucity of studies on the automatic identification of Down syndrome with facial recognition technologies, especially using deep convolutional neural networks. Here, we developed a Down syndrome identification method utilizing facial images and deep convolutional neural networks, which quantified the binary classification problem of distinguishing subjects with Down syndrome from healthy subjects based on unconstrained two-dimensional images. The network was trained in two main steps: First, we formed a general facial recognition network using a large-scale face identity database (10,562 subjects) and then trained (70%) and tested (30%) a dataset of 148 Down syndrome and 257 healthy images curated through public databases. In the final testing, the deep convolutional neural network achieved 95.87% accuracy, 93.18% recall, and 97.40% specificity in Down syndrome identification. Our findings indicate that the deep convolutional neural network has the potential to support the fast, accurate, and fully automatic identification of Down syndrome and could add considerable value to the future of precision medicine.

## 1. Introduction

Down syndrome is one of the most common genetic syndromes caused by a chromosome 21 abnormality with a prevalence of 1:1000–1100 worldwide [1]. Patients with Down syndrome are typically associated with characteristic facial features, physical growth delays, mild to moderate intellectual disabilities [2,3,4,5,6,7], and an increased risk of complications for respiratory and hearing problems, as well as heart defects [5,8]. Early diagnosis is necessary to prevent the occurrence of potential health problems and to benefit patients with lifelong healthcare involving physical, speech, cardiac, and neurological therapies [9].

The diagnosis of Down syndrome can be conducted during pregnancy or after birth [10,11]. Screening for Down syndrome is recommended as universally offered to pregnant women and is a critical component of antenatal care [12,13,14]. After birth, Down syndrome can be identified by the presence of some typical facial characteristics [5,6,7,15,16]. Some of these features include upslanted palpebral fissures, a flat nasal bridge, widely spaced eyes, a protruding tongue, and small ears and nose [4,15,17]. A chromosome test, called a karyotype test, should be performed to confirm this diagnosis [1,18]. However, the recognition of nonclassical presentations of Down syndrome is constrained by clinical experts’ prior experience. Chromosome tests are expensive, complicated, and time-consuming, and many remote health organizations have no access to these technologies. Therefore, the utilization of computerized systems among health professionals is becoming increasingly essential.

Recent advances in computer vision and deep learning present the opportunity for development in many fields. The performance of tasks such as object detection, localization, recognition, and segmentation based on public datasets has dramatically improved in recent years [19,20,21,22,23,24]. One of the main types of deep learning methods, the deep convolutional neural network (DCNN), applies a series of layers, including convolution layers, pooling layers, and fully connected layers, with thousands or even millions of trainable parameters that are continuously updated with a backpropagation algorithm to minimize the loss between the outputs and targets during the training process [25,26]. In medicine, deep learning has demonstrated significant advantages in disease diagnosis and lesion segmentation due to its powerful capacity for feature extraction [27,28,29]. The distinctive facial characteristics of Down syndrome might also provide an opportunity for automatic identification. In recent years, few studies have been undertaken to identify cases of Down syndrome using two-dimensional or three-dimensional facial images. A study proposed by Zhao et al. [9] used facial geometric and texture biomarkers for Down syndrome identification with 2D facial images. The facial characteristics were presented with geometric features based on facial anatomical landmarks, local texture features based on contourlet transform, and local binary patterns. The normal and abnormal cases were discriminated by machine learning (ML) methods, including support vector machine (SVM) and k-Nearest Neighbors (KNN). However, this method needed to manually extract geometric features from patients, and the dataset only involved 24 Down syndrome cases and 24 normal cases. To our knowledge, there have not been any published reports related to the fully automatic identification of Down syndrome with facial recognition technologies, especially using DCNN.

In the clinic, physicians normally need to distinguish Down Syndrome from healthy subjects. Hence, we developed a Down syndrome identification method utilizing facial images and deep convolutional neural networks which quantified a binary classification problem of distinguishing Down syndrome from healthy. In this study, we investigate a novel method to identify Down syndrome, using only facial images with DCNN. The proposed DCNN training algorithm involves three steps: image preprocessing, general facial recognition network training, and Down syndrome identification network training. The image preprocessing method includes four main steps: image enhancement, facial detection, facial cropping, and image resizing. The large-scale facial recognition dataset was randomly split into training and testing datasets for the general facial recognition network training. Transfer learning was applied to fine-tune the Down syndrome identification network with two output classes (Down syndrome and healthy). The method’s accuracy, recall, specificity, $F_{1}$ score, Matthias Correlation Coefficient (MCC) scores, and quadratic weight κ were measured. Our method provides a fast, accurate, and fully automatic clinical tool to identify Down syndrome with great potential to support the remote identification of genetic disorders with full automation for possible application in precision medicine.

Our main contributions include the following:

- Developing a novel fully automatic Down syndrome identification method based on unconstrained 2D facial images. Unlike traditional chromosome tests for Down syndrome diagnosis, which are expensive, complicated, and time-consuming, the utilization of DCNN with facial images could simplify the identification procedure considerably. To our knowledge, this is the first time that DCNN has been applied to Down syndrome identification with facial images.
- Utilizing DCNN for Down syndrome identification with high accuracy outperforms the state-of-the-art method reported by previous studies. In addition, we demonstrate that the identification results are based on the facial characteristics of Down syndrome patients, which is illustrated in the extracted feature maps.
- Demonstrating that the algorithm applied in DCNN training, including image preprocessing and transfer learning, contributes to the performance improvement of the DCNN in Down syndrome identification, which is illustrated in the ablation experiment.

Our paper is organized as follows. Section 2 describes the proposed methods, including the Down syndrome identification pipeline, image preprocessing, dataset, principles, training details, and model evaluation protocol. Section 3 presents the results, summarizing the performance evaluation, performing the ablation experiment, and comparing our method with other state-of-the-art methods. Finally, Section 4 and Section 5 provide the discussion and conclusions as well as possible future work.

## 2. Materials and Methods

This section describes the technology related to the Down syndrome identification system, including the Down syndrome identification pipeline, image preprocessing, dataset, principles, and training details. Down syndrome identification is a kind of binary classification problem. Our goal was to form a convolutional neural network model that maps an unconstrained 2D RGB facial image as the input and outputs the result (Down syndrome or healthy) and the probability of having Down syndrome.

### 2.1. Down Syndrome Identification Pipeline

The diagram for the proposed training algorithm contains three main steps: image preprocessing, the general facial recognition network training, and the Down syndrome identification network training. A DCNN was needed to extract these features for prediction since the faces of Down syndrome have subtle differences from the healthy. As the Down syndrome image dataset was relatively small compared to the general facial recognition dataset, which easily caused an overfitting problem in the training process, transfer learning was applied for the application of general facial recognition knowledge to the specific problem.

All images from the datasets were preprocessed before training, involving image enhancement, facial detection, facial cropping, and image resizing. The DCNN was first trained on a large-scale database to obtain a general facial recognition network. After this, the final fully connected layer of DCNN was modified to fit the needs of Down syndrome identification. This involved knowledge transfer from the source domain (facial recognition) to the target domain (Down syndrome identification) [30]. In this process, the network was refined for Down syndrome identification, outputting the probability of having Down syndrome by inputting a facial image.

**Figure 1:**
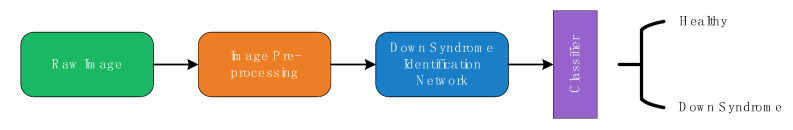
The identification process for the real clinic situation.

The model operation pipeline for a real clinical situation is illustrated in Figure 1. A raw frontal facial image that was not in the training dataset was preprocessed and input to the Down syndrome identification network. The output was the similarity of the input image with Down syndrome and healthy faces with the training dataset, indicating the possibility of having Down syndrome. After passing the classifier, the pipeline output the final Down syndrome identification result.

### 2.2. Image Preprocessing

The image preprocessing method involved four main steps: image enhancement, facial detection, facial cropping, and image resizing. As the raw images were taken in real life with a large variance in exposure and contrast, image enhancement was needed for superior detection of the facial area in the second step. In addition, the impact of irrelevant factors such as exposure, contrast, and different cameras could be eliminated by image enhancement, which helped the network concentrate more on facial features. We applied an auto image adjustment method using the MATLAB image processing toolbox. The images were adjusted by mapping the values of the input intensity image to the new value, in which 1% of the values were saturated with low and high intensities of the input data.

We then used a face detector to detect the face’s position to identify the facial area from the background. Since unconstrained 2D facial images had differences in face sizes, expressions, and backgrounds, a robust and highly accurate face detector was needed. The deep learning method performs well in facial detection. Hence, we adopted multitask cascaded convolutional neural networks to obtain the face’s position [31]. The original networks were adjusted to fit our needs and were applied to the facial images.

After obtaining the position of face, the face was then cropped from the enhanced image as the input to the DCNN to make the Down syndrome identification network more concentrated on the information from faces rather than the background to improve its accuracy. Finally, as the cropped facial images had differences in size, they were resized to 192 × 192 × 3 to meet the requirements of the network input.

### 2.3. Dataset

A large-scale facial recognition database was adopted to train the general facial recognition network. The images from publicly available CASIA-WebFace datasets were screened manually, and those with poor image quality or insufficient images of the subjects were discarded [32]. Finally, 493,750 images with 10,562 subjects were split into a training dataset (90%) and testing dataset (10%) and applied to pretraining of the Down syndrome identification network.

Positive and negative Down syndrome facial images were applied to fine-tune the networks by capturing phenotypic information. Frontal Down syndrome facial images were all obtained from public databases, involving Face2Gene (http://www.fdna.com/face2gene/) and the elife database [33]. The resolution, illumination, and face pose varied. All the images were labelled to indicate their diagnostic conditions and were authorized again by the professionals to ensure that the correct label was used. Invalidated or duplicated images were discarded. As the Down syndrome identification system should be available for all ages, the negative cohort images were healthy people selected from the aged based database Adience [34,35]. The selection covered all ages, from infants to adults, to enhance the network’s robustness. The entire negative cohort was authorized by the professionals to ensure that the subjects did not include Down syndrome facial characters. Finally, 148 Down syndrome images with 127 subjects were used as a positive cohort and 257 images as a negative cohort. All images had labels to indicate their status. The whole dataset involving Down syndrome and healthy facial images was separated into a training dataset (70%) and a testing dataset (30%). The training dataset was used for transfer learning of the Down syndrome identification network, and the testing dataset was used for further testing of the network performance and selection of the best model.

### 2.4. Principle

DCNN is the most common type of neural network for image classification. It is also a kind of feedforward neural network that performs well in facial recognition. The DCNN that we applied for Down syndrome identification contains an image input layer, multiple hidden layers (including convolutional layers, pooling layers, and fully connected layers), and an output layer (softmax).

For hidden layers, the input and output were linked via multiple neurons, defined as follows [25]:

(1) $$y=g\left(b+\overset{m}{\underset{k=1}{\sum }}w_{i}z_{i}\right)$$

where y represents the output of a neuron, $z_{i}$ represents the input, and $w_{i}$ and b are the parameters in the neural networks that were obtained by training. g is the activation function.

The selection of the activation function contributed to the training speed and final identification accuracy. The rectified linear unit (ReLU) performed well in facial recognition networks as the activation function and was selected as the activation function [36].

The pooling layer can reduce the dimensions of the data by computing the overall characteristics of the rectangular regions and by replacing the detailed output by their overall statistical features, which can reduce the cost of computations and can increase the training and identification speed. Thus, the max-pooling layers were applied between every two convolutional layers.

The softmax function was used to calculate the probability distribution of the event over different classes, which normalized the sum possibility of every class to 1. The probability of turning on class i is a softmax function of its direct ancestral fully connected layer neurons, where exactly one unit is allowed to be active and the probability is reconstructed [25]. A higher score indicates a higher possibility, which is the final classification result of the input image. Hence, the softmax function was used as the final stage of classifier.

As the network is used for classification, the final output indicates the possibility in each class, which generally uses cross-entropy as the loss function [37,38]. To reduce overfitting, a regularization term (weight decay) for the weights is added to the cross-entropy loss [39,40,41]. Hence, cross-entropy with $L_{2}$ regularization is applied as a loss function $Loss_{R}$, which takes the following form:

(2) $$Loss_{R}=-\overset{N}{\underset{i=1}{\sum }}\overset{K}{\underset{j=1}{\sum }}t_{ij}log\left(y_{ij}\right)+\frac{1}{2}\times \lambda \times w^{T}w$$

For the cross-entropy term, N is the number of samples, K is the number of classes, $t_{ij}$ indicates that the $i^{th}$ sample belongs to the $j^{th}$ class (which is 1 when labels correspond and 0 when they are different), and $y_{ij}$ is the output of sample i for class j, which is the output value of the softmax layer. For the cross-entropy term, w is the learned parameters in every learned layer and λ is the regularization factor (coefficient).

As Down syndrome identification is a binary classification problem, cross-entropy with $L_{2}$ regularization loss can be defined as follows:

(3) $$Loss_{B-R}=-t_{1}log\left(y\right)-\left(1-t_{1}\right)log\left(y\right)+\frac{1}{2}\times \lambda \times w^{T}w$$

where $t_{1}=1$ when true and predict labels match and where $t_{1}=0$ when the labels are different. y is the output of the similarity between the input image and the Down syndrome training dataset.

### 2.5. Network Architecture

The raw input images were processed by cascade convolutional neural networks between the input and the output. The first stage was to extract and crop the facial area. The second stage was Down syndrome identification.

For facial detection in the first stage, the convolutional neural network (CNN) architecture was based on multitasking cascaded convolutional neural networks, which are unified cascaded CNNs that use multitask learning [31]. The CNNs included P-Net, R-Net, and O-Net, which detected the facial area after the images were processed by three cascaded CNNs in order. The pretrained model was then modified for facial area detection and extraction.

**Figure 2:**
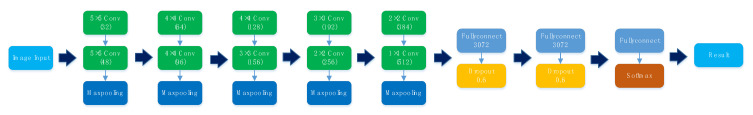
The architecture of the deep convolutional neural network (DCNN). The horizontal big arrows represent the data flow across successive functional blocks of the network, whereas the vertical small arrows represent the data flow inside each block.

The DCNN applied to Down syndrome identification in the second stage contained eleven learned layers, involving eight convolutional layers that could be divided into four inception blocks and three fully connected layers. The detailed architecture is summarized in Figure 2.

As the patients’ faces had slight differences with healthy people’s faces, in order to improve the final prediction accuracy, a large image input was adopted. Since the receptive field of every convolutional layer contributed to the final accuracy of the classification network identification, maintaining the receptive field in every convolutional layer was needed. One way was to increase the kernel size, which increased the total calculation costs and the possibility of overfitting. Hence, the concept of the inception model was applied to reduce the cost of computation, which factorized one large kernel into a multilayered network of smaller kernels [42]. For example, in the first two convolutional layers, two 5 × 5 kernels were used as a replacement for a 9 × 9 convolutional kernel, which significantly reduced the number of parameters. The multilayered network also used more activation functions, which allowed for more disentangled features. The number of filters in every layer was designed based on a factor of 1.5. The 512 1 × 1 kernels in the final convolutional layer were applied to combine information from the different filters and to increase the dimensions of the feature map, which was inspired by GoogLeNet [42].

There were 10 convolutional layers in total, which were all activated by ReLU. Every two convolutional layers formed a block, followed by a max-pooling layer to reduce computations. The two fully connected layers had 3072 neurons each, following the last max-pooling layer, each with a dropout factor of 0.6 for regularization [40,43]. The output of the last fully connected layer was input to the softmax layer, which produced a distribution of the numbers of class labels, in which 10,562 classes were for the general facial recognition network and 2 were for the Down syndrome identification network (i.e., Down syndrome and healthy). The result was a class label with maximum probability.

### 2.6. Training Details

The training goal of the DCNN was to maximize the multinomial logistic regression objective, which is equivalent to maximizing the average log-probability when the predictions match the true labels. When training the DCNN, the true labels were provided as part of the input; for classification problems, the labels were one-hot vectors that represented the true classes, which can be described as follows:

(4) $$V=\left[x_{0},x_{1},\ldots ,x_{n}\right]$$

where V represents the input label vector, $x_{i}\;\left(i\in \left[1,n\right]\right)$ represents the true class of the image, and n represents the class number. When the image was in the i class, $x_{i}=1$ and $x_{k}=0\;\left(k!=i\right)$.

For general facial recognition network training, the large-scale facial recognition dataset was randomly divided into a training dataset (90%) and testing dataset (10%). The output classes were set to match the class numbers (10,562). The network was trained for 50 epochs, with the training dataset shuffled every epoch. The learning rate was set as $10^{-4}$, with a learning rate drop factor of 0.9 every 6 epochs. The testing showed that the loss became stable at around 6 epochs with a constant learning rate and that a learning rate drop factor of 0.9 every 6 epochs could significantly increase the training speed. The parameters of w in (1) were initialized using the Glorot initializer, and those of b were initialized with zeros [44]. Adam was adopted as an optimizer, with a $10^{-4}$ weight decay for $L_{2}$ regularization to avoid overfitting [45].

For the Down syndrome identification network training, which applied transfer learning to fine-tuning the network architecture, the final fully connected layer and the classifier were modified to match the number of classes (Down syndrome and healthy), with a learning rate bias of 25 for both parameters (w and b in Equation (1)). All trainable parameters were relearned in the transfer learning process. Adam was adopted as the optimizer with a learning rate of $10^{-4}$. A $10^{-4}$ weight decay for $L_{2}$ regularization was also applied to avoid overfitting. The network was trained for 10 epochs to reach high accuracy with a batch size of 16. All the parameters mentioned above were determined using the grid search method for the best identification accuracy on the testing dataset.

Augmentation was proven to have a significant impact on the final accuracy of the network for the reduction of overfitting [21,46,47]. For general facial recognition network training, the training dataset was randomly augmented by rotation with a range of 10 degrees (in a normal distribution) as well as a vertical and horizontal shift (a shift with a range of 8 pixels) and a horizontal flip in every epoch. In the fine-tuning stage, to make the network more strongly focus on the details of the images in the small dataset with fewer training epochs, the augmentation was set to be mild, with a 1 degree rotation range (in a normal distribution), a vertical and horizontal shift range of 3 pixels, and a random horizontal flip in every epoch.

## 3. Results

The Down syndrome identification method we proposed was applied using MATLAB, with the deep learning toolbox for network training and the image processing toolbox for image preprocessing [48]. The network was trained using a single Nvidia graphics processing unit (GPU) with compute unified device architecture (CUDA) and Nvidia CUDA deep neural network library (cuDNN) enabled.

After training, the network was tested using positive and negative Down syndrome facial images mentioned above. The results were reported by their accuracy, recall, specificity, $F_{1}$ score, Matthias Correlation Coefficient (MCC) scores, and quadratic weight κ. In addition, the confusion matrix and the active parts of the feature maps in every convolutional layer were visualized for further evaluation of the DCNN. The AUPRC (area under the precision-recall curve) and AUROC (area under the receiver operating characteristic curves) were also measured alongside the report of the precision-recall curve (PRC) and receiver operating characteristic (ROC) curves. The network was also compared with the state-of-the-art method to illustrate its performance.

### 3.1. Down Syndrome Identification Results

The accuracy, recall, specificity, $F_{1}$ score, and MCC scores were important factors for measuring the performance of the network. Their definitions are as follows:

(5) $$Accuracy=\frac{TP+TN}{TP+TN+FP+FN}$$

(6) $$Recall=\frac{TP}{TP+FN}$$

(7) $$Specificity=\frac{TN}{TN+FP}$$

(8) $$Precision=\frac{TP}{TP+FP}$$

(9) $$F_{1}=2*\frac{Precision\times Recall}{Precision+Recall}$$

(10) $$MCC=\frac{TP\times TN-FP\times FN}{\sqrt{\left(TP+FP\right)\left(TP+FN\right)\left(TN+FP\right)\left(TN+FN\right)}}$$

where TP, TN, FP, and FN correspond to true positive, true negative, false positive, and false negative, respectively.

The performance of the proposed method is described in Table 1. The Down syndrome identification network reached an accuracy of 95.87% with 93.18% sensitivity and 97.40% specification.

Previous studies have shown that the choice of optimizer in the network training has a significant impact on the final network performance [45]. Hence, different optimizers in the fine-tuning stage of the network were tested as a grid search for the best algorithm. The results are shown in Table 1. The initial learning rates were set using the grid search method for the optimizer to reach the highest accuracy. For Adam, the rate was $10^{-4}$; for stochastic gradient descent with momentum (SGDM), it was $5\times 10^{-6}$ with 0.9 momentum; and for root mean square propagation (RMSProp), it was $2\times 10^{-5}$ [49]. All other parameters, including the batch size, augmentation, and fully connected layer learning rate bias were set to be the same. The network using an Adam optimizer reached the highest quadratic weight κ on the testing dataset compared to the others. Hence, Adam was chosen as the optimizer for the fine-tuning stage of the DCNN.

**Figure 3:**
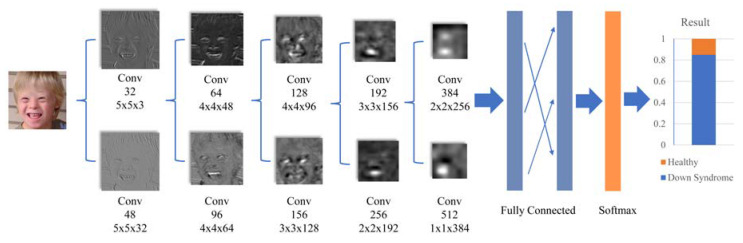
Down syndrome identification for a facial image (The original facial image is available at https://en.wikipedia.org/wiki/Down_syndrome). The horizontal big arrows represent the data flow across successive functional blocks of the network, whereas the small arrows represent the connections between the fully connected layers.

The feature maps of every convolution layer were extracted, which help with the understanding of the Down syndrome identification network. As there were multiple feature maps in every convolution layer, the brightest parts of every feather map were combined in each layer and turned into grayscale, representing the activation parts of the image. The bright parts indicated that it was activated (which was the information the network concentrated on). As shown in Figure 3, the result was determined mostly by the information from the eyes, nose, and mouth, which presented significant differences between the Down syndrome patients and healthy individuals. The similarities between the artificial and DCNN Down syndrome identification patterns indicate that the proposed method successfully obtained the medical characteristics of the Down syndrome patients’ faces.

The feature maps from the general facial recognition network were also extracted and compared with those of Down syndrome identification network. The results showed a considerable difference in deep convolutional layers (deeper than the third layer). The areas of the eyes, nose, and mouth in feature maps extracted from Down syndrome identification network had higher values than the general face recognition network, which meant the information from those areas were more concentrated. These were the main facial medical characteristics of Down syndrome patients, and the Down syndrome identification network obtained knowledge by transfer learning.

### 3.2. Ablation Experiment

To demonstrate the importance of image preprocessing and transfer learning, an ablation experiment was conducted. Based on the proposed method, the network was trained and tested using (1) the proposed method, which applied image preprocessing and transfer learning; (2) the proposed method without image preprocessing in training and testing; (3) the proposed method without using transfer learning; and (4) the proposed method without transfer learning or image preprocessing in training and testing. All other training and testing parameters remained the same.

The testing results are shown in Table 2. As shown in the ablation results, the network with image preprocessing and transfer learning achieved a considerable performance gain compared to the control groups. Without image preprocessing, the network could not concentrate on facial information, since the backgrounds occupied more space than the faces themselves. Moreover, the images had differences in their exposure and contrast, which was also detrimental to the final performance. Without transfer learning, the network easily experienced overfitting and could not extract appropriate facial features for Down syndrome identification.

### 3.3. Comparison with State-of-the-Art Method

There are few studies related to Down syndrome identification using facial images with computer vision. The state-of-the-art involves applying ML methods, including SVM and KNN, for Down syndrome identification with manually processed facial images [9]. Hence, we compared the performance of the proposed method with that of KNN and SVM [50,51,52].

All the methods were tested using the same testing set. The accuracy, recall, specificity, $F_{1}$ score, MCC scores, and quadratic weight κ of the results were reported, as shown in Table 3. The proposed method outperformed KNN and SVM in every aspect.

**Figure 4:**
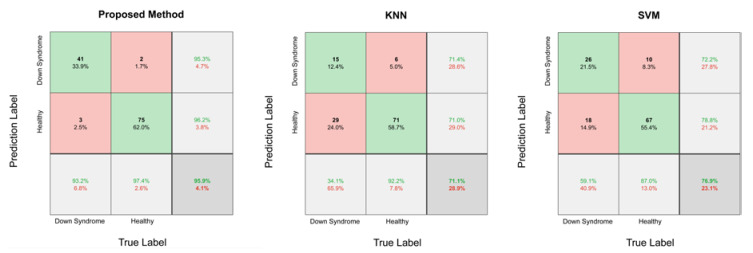
Confusion matrix of the proposed method, k-Nearest Neighbors (KNN), and support vector machine (SVM). Green squares denote the correctly classified cases, red squares denote the incorrectly classified cases, gray squares denote the identification results in rows/columns, and deep grey ones denote the Down syndrome identification results. Green numbers represent the correct rates, and red numbers represent the error rates.

Comparisons of the confusion matrixes are shown in Figure 4. The testing dataset contained 44 Down syndrome face images and 77 health images. The method we proposed correctly classified 116 images with only 5 prediction errors, mainly due to facial directions or the characteristics of the eyes. Based on failed case analyses of the proposed method, the DCNN is likely to have a bias when individuals do not face directly toward the camera. For Down syndrome patients, the DCNN had a bias with a higher probability when the characteristics of their eyes were not distinctive enough.

**Figure 5:**
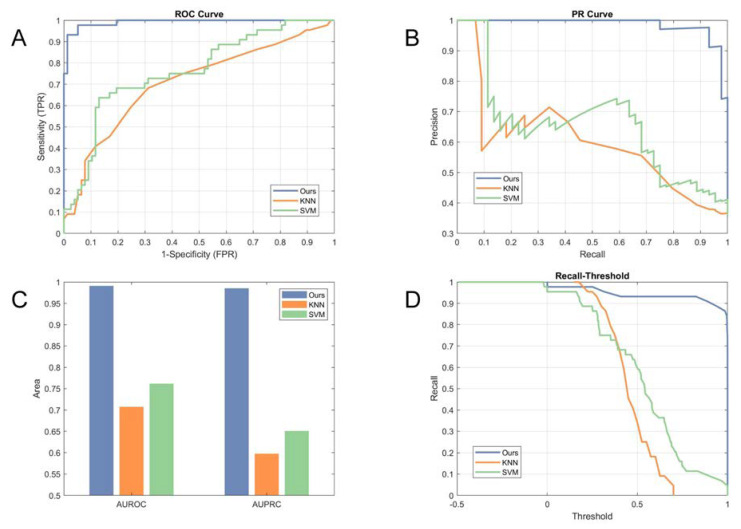
Comparison with state-of-the-art methods: (**A**) Comparison in a receiver operating characteristic (ROC) curve; (**B**) comparison in a precision-recall curve (PR curve); (**C**) comparison in area under the receiver operating characteristic curves (AUROC) and area under the precision-recall curve (AUPRC); and (**D**) illustration in the recall of methods vs. varying normalized score thresholds.

The PRC, AUPRC, ROC, and AUROC were adopted to measure the performance gains of the proposed method and to indicate the effectiveness of screening. PRC and ROC were utilized to evaluate the performance of the algorithms, with the AUPCR and AUROC applied to characterize the level of classification. The comparisons using the ROC and PRC are illustrated in Figure 5A,B, respectively. The experimental results show that the proposed method substantially improved the performance of Down syndrome identification, with an AUPRC of 0.9854 and an AUROC of 0.9909 compared to 0.5978 and 0.7073 for the KNN and 0.6508 and 0.7618 for the SVM, as shown in Figure 5C.

To indicate the influence of thresholds on the identification results, we plotted recall as a function of the thresholds predicted by the proposed algorithm and the other methods, as shown in Figure 5D. Our proposed method had a large recall, even for a high threshold, which indicates that the Down syndrome network had higher confidentiality in most of the Down syndrome identification results compared with the state-of-the-art methods. By combining the information of the sensitivity-threshold curve with the PRC and ROC, every point on the PRC and ROC could demonstrate its correlated threshold.

## 4. Discussion

Our study presents a novel facial recognition method that uses DCNN to identify Down syndrome automatically from 2D facial images in a large-scale facial recognition dataset. We presented how this framework is able to accurately generalize for a specific problem and indicated how a binary model can be trained to identify Down syndrome. Finally, we found that the DCNN achieved 95.87% accuracy, 93.18% recall, and 97.40% specificity in identifying Down syndrome. The importance of transfer learning and image preprocessing was illustrated in the ablation experiment. In addition, we compared the performance of the proposed method with that of state-of-the-art methods (KNN and SVM) and found that the proposed method obtained higher precision in the same recall and always had a larger recall whenever the value was of 1-specificity. This finding indicates that, by using only facial images with a pretrained general facial recognition model, the DCNN can achieve highly accurate results in identifying Down syndrome.

The identification of Down syndrome has many similarities to classic facial recognition. However, the development of syndrome recognition in practice is challenging for several reasons, including limited data, ethnic differences, subtle facial patterns, and ethical problems. In the case of Down syndrome, it is impossible to gather a large dataset due to the relatively few people with this syndrome. Hence, image preprocessing and transfer learning were applied in the training procedure for the DCNN to avoid a possible overfitting problem due to a limited dataset.

Currently, DCNN is one of the most common solutions for computer vision tasks and can be used for feature extraction and classification, as presented in our study. It is a robust classification model when provided enough labelled data of the target domain for training and is especially adept at observing data with slight differences. Patients with Down syndrome have distinctive but subtle facial features, which provide the possibility for automatic identification. Based on the literature review, no attempt has been undertaken to detect Down syndrome using DCNN with facial images due to the highly imbalanced data and similar prototypes between patients and healthy individuals.

In our study, Down syndrome subjects were detected with 95.87% accuracy. This encouraging finding indicates that our method has great potential to support the DCNN-based identification of Down syndrome from facial images. Furthermore, our method should be validated for its operational value across the healthcare system by broadening the dataset and by verifying its accuracy in the identification of genetic syndromes.

## 5. Conclusions

In this paper, we proposed an automatic identification of Down syndrome using digital facial images with DCNN. The proposed DCNN training algorithm contains three steps: image preprocessing, general facial recognition network training, and Down syndrome identification network training. The performances of the proposed method were measured in terms of its accuracy, recall, and specificity. The experimental results show that Down syndrome was detected with 95.87% accuracy and 93.18% recall. The results indicate that our method has great potential to support the automatic identification of Down syndrome from facial image data and would be useful for the early screening and prevention of disease progression. In future studies, we will investigate various genetic diseases that affect facial features and will apply genome sequencing data to assist in clinical diagnosis and to open a new pathway in the field of precision medicine.

## Figures and Tables

**Table 1:** Performance of the DCNN in identifying Down syndrome.

Optimizer	Accuracy	Recall	Specificity	Precision	$F_{1}$	MCC	κ
Adam	**95.87%**	**93.18%**	**97.40%**	**95.34%**	**95.24%**	**91.04%**	**91.03%**
SGDM	91.74%	90.91%	92.21%	86.96%	91.56%	82.37%	82.32%
RMSPROP	95.04%	95.45%	94.81%	91.30%	95.13%	89.44%	89.39%

The proposed method is made bold, and the best results are shown in blue.

**Table 2:** Performance of the DCNN in ablation experiment.

Ablation Experiments	Accuracy	Recall	Specificity	Precision	$F_{1}$	MCC	κ
(1)	**95.87%**	**93.18%**	**97.40%**	**95.34%**	**95.24%**	**91.04%**	**91.03%**
(2)	57.85%	97.73%	35.06%	46.24%	62.78%	37.40%	26.47%
(3)	83.47%	77.27%	87.01%	77.27%	77.27%	64.29%	64.29%
(4)	68.60%	86.36%	58.44%	54.28%	66.66%	43.65%	39.77%

(1) The proposed method, which applied image preprocessing and transfer learning; (2) the proposed method without image preprocessing in training and testing; (3) the proposed method without using transfer learning; (4) the proposed method without transfer learning or image preprocessing in training and testing. The proposed method is made bold, and the best results are shown in blue.

**Table 3:** Performance comparison with state-of-the-art methods.

Method	Accuracy	Recall	Specificity	Precision	$F_{1}$	MCC	κ
**Ours**	**95.87%**	**93.18%**	**97.40%**	**95.34%**	**95.24%**	**91.04%**	**91.03%**
**KNN**	71.07%	34.09%	92.21%	71.43%	46.15%	33.40%	29.62%
**SVM**	76.86%	59.09%	87.01%	72.22%	65.00%	48.51%	47.97%

The proposed method is made bold, and the best results are shown in blue.
